# Proteome Analyses of Cellular Proteins in Methicillin-Resistant *Staphylococcus aureus* Treated with Rhodomyrtone, a Novel Antibiotic Candidate

**DOI:** 10.1371/journal.pone.0016628

**Published:** 2011-02-04

**Authors:** Wipawadee Sianglum, Potjanee Srimanote, Wijit Wonglumsom, Kanokwan Kittiniyom, Supayang P. Voravuthikunchai

**Affiliations:** 1 Department of Microbiology and Natural Products Research Center, Faculty of Science, Prince of Songkla University, Songkla, Thailand; 2 Graduate Study, Faculty of Allied Health Sciences, Thammasat University, Pathumtanee, Thailand; 3 Department of Clinical Microbiology, Faculty of Medical Technology, Mahidol University, Nakhon Pathom, Thailand; University of Cambridge, United Kingdom

## Abstract

The ethanolic extract from *Rhodomyrtus tomentosa* leaf exhibited good antibacterial activities against both methicillin-resistant *Staphylococcus aureus* (MRSA) and *S. aureus* ATCC 29213. Its minimal inhibitory concentration (MIC) values ranged from 31.25–62.5 µg/ml, and the minimal bactericidal concentration (MBC) was 250 µg/ml. Rhodomyrtone, an acylphloroglucinol derivative, was 62.5–125 times more potent at inhibiting the bacteria than the ethanolic extract, the MIC and MBC values were 0.5 µg/ml and 2 µg/ml, respectively. To provide insights into antibacterial mechanisms involved, the effects of rhodomyrtone on cellular protein expression of MRSA have been investigated using proteomic approaches. Proteome analyses revealed that rhodomyrtone at subinhibitory concentration (0.174 µg/ml) affected the expression of several major functional classes of whole cell proteins in MRSA. The identified proteins involve in cell wall biosynthesis and cell division, protein degradation, stress response and oxidative stress, cell surface antigen and virulence factor, and various metabolic pathways such as amino acid, carbohydrate, energy, lipid, and nucleotide metabolism. Transmission electron micrographs confirmed the effects of rhodomyrtone on morphological and ultrastructural alterations in the treated bacterial cells. Biological processes in cell wall biosynthesis and cell division were interrupted. Prominent changes including alterations in cell wall, abnormal septum formation, cellular disintegration, and cell lysis were observed. Unusual size and shape of staphylococcal cells were obviously noted in the treated MRSA. These pioneer findings on proteomic profiling and phenotypic features of rhodomyrtone-treated MRSA may resolve its antimicrobial mechanisms which could lead to the development of a new effective regimen for the treatment of MRSA infections.

## Introduction

*Staphylococcus aureus* is well-evidenced as a major human pathogen. The organism commonly involves in skin and soft tissue infections such as pimples, boils, furuncles, cellulites, folliculitis, impetigo, carbuncles, scalded skin syndrome, and abscesses. In addition, it can cause some serious infections including bacteremia, pneumonia, acute endocarditis, meningitis, osteomyelitis, toxic shock syndrome, and fatal invasive diseases [1]. The emergence and spread of methicillin-resistant *S. aureus* (MRSA) originated in the 1960s as an important clinical and epidemiological problem in hospital environments. Additionally, *S. aureus* has been a crucial cause of community-acquired infections which may result in morbidity and mortality [2], [3]. The introduction of glycopeptide antibiotics, a last resort to treat such infections, was followed by the isolation of either vancomycin-intermediate *S. aureus* (VISA) or vancomycin-resistant *S. aureus* (VRSA) [4], [5].

Several active researches have documented new antibiotics and semi-synthetic analogs with improved antimicrobial properties [6], [7], [8]. Various plants worldwide have been used in traditional medicine as alternative treatments of bacterial infections [9], [10], [11]. Rhodomyrtone, an acylphloroglucinol derivative isolated from the leaf of *Rhodomyrtus tomentosa* (Aiton) Hassk., has been briefly reported to produce antibacterial effects against *Escherichia coli* and *S. aureus* [12]. From our preliminary studies, the data suggested that the ethanolic extract of *R. tomentosa* and rhodomyrtone presented strong antimicrobial activity against a wide range of Gram-positive bacteria such as *Bacillus subtilis*, *Enterococcus faecalis*, *S. aureus*, *S. epidermidis*, and *Streptococcus* spp. [13], [14], [15]. In addition, rhodomyrtone also exhibited significant antimicrobial activity against *S. epidermidis* biofilm-forming and *S. pneumoniae* capsule-producing strains [15].

In recent years, two dimensional gel electrophoresis (2DE) reference maps of cellular or extracellular proteins from various *S. aureus* strains in different growth conditions have been established [16], [17], [18]. The identification of cell surface, cell membrane, and cytoplasmic proteome map of *S. aureus* provides essential tool for better understanding of biological, pathological, and physiological significance of the bacteria. Furthermore, using 2DE separation technique combined with tryptic peptide mass mapping via mass spectrometry, the global view of the synthesis and distribution of various *S. aureus* protein networks was obtained [16], [19]. To provide insights into the antibacterial mechanisms of this potential antibacterial drug, proteomic technologies were used to investigate the effects of rhodomyrtone on protein expression in treated MRSA cells. Achievements in protein analysis have been acquired by the combination of mass spectrometry techniques and bioinformatic tools. The effects of rhodomyrtone on morphological and ultrastructural changes in the treated staphylococcal cells were further elucidated using transmission electron microscopy.

## Results and Discussion

### Determination of antibacterial activity of ethanolic extract of *R. tomentosa* and rhodomyrtone

The efficacy of most current antibiotics for treatment of staphylococcal infections has become quite limited due to the occurrence of multiple resistant strains [20]. In very recent studies, several new compounds from natural products and their derivatives have been extensively studied to treat resistant *S. aureus* [21], [22], [23]. A clinical MRSA isolate, NPRC 001R, was found to be resistant to several classes of antibiotics including *β*-lactam, aminoglycoside, tetracycline, macrolide, fluoroquinolone, and clindamycin. Therefore, it has been used as a representative isolate throughout this study. In order to assess the antibacterial potency of ethanolic extract of *R. tomentosa* and rhodomyrtone on *S. aureus*, susceptibility test using broth microdilution was employed. The ethanolic extract exhibited good antibacterial activities against both MRSA and *S. aureus* ATCC 29213. Its minimal inhibitory concentration (MIC) values were 31.25–62.5 µg/ml, and the minimal bactericidal concentration (MBC) was 250 µg/ml. Rhodomyrtone was 62.5–125 times more potent at inhibiting both MRSA and *S. aureus* ATCC 29213 than the crude extract, its MIC and MBC values were 0.5 µg/ml and 2 µg/ml, respectively.

### Time-kill assay

**Figure 1:**
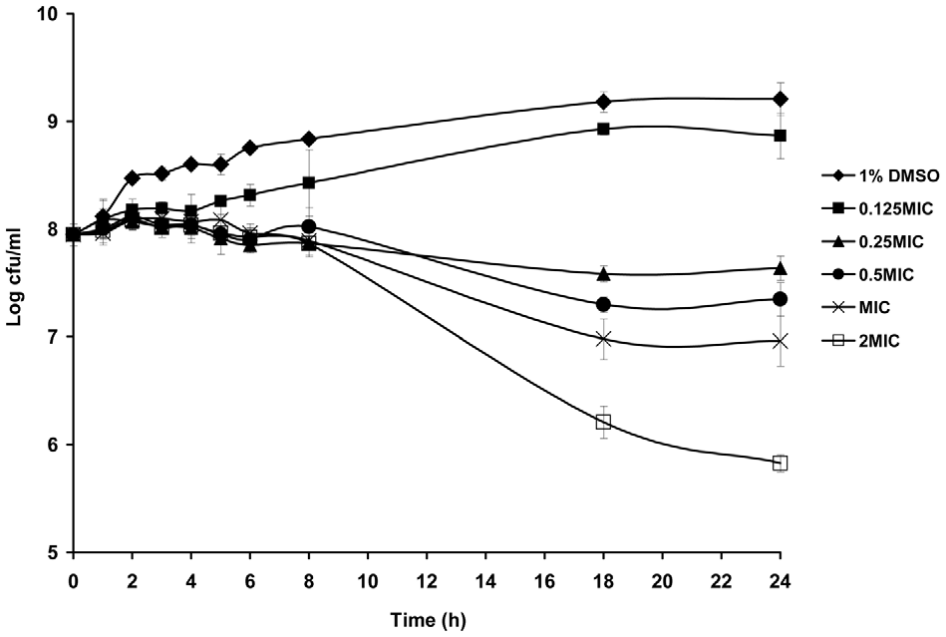
Time-kill curves of rhodomyrtone against MRSA. Viability was counted at the indicated time points by serial dilution plating. Each point represented the mean of log_10_ ± standard deviations of three different experiments performed in duplicate.

Time-kill kinetic was carried out with MRSA isolates. Viable cell counts of the bacteria after treatment with rhodomyrtone were similar. The results of the time-kill assay of a representative isolate were shown in Figure 1. Within 4–5 h, the bacterial growth was stable at initial inoculum after treated at 0.125, 0.25, 0.5, 1, and 2MIC of rhodomyrtone. The number of viable cells after exposure to MIC and 2MIC of rhodomyrtone decreased by two log-fold, compared to the control within 18 h.

### Protein variation between cells cultured in the presence or the absence of rhodomyrtone

**Figure 2:**
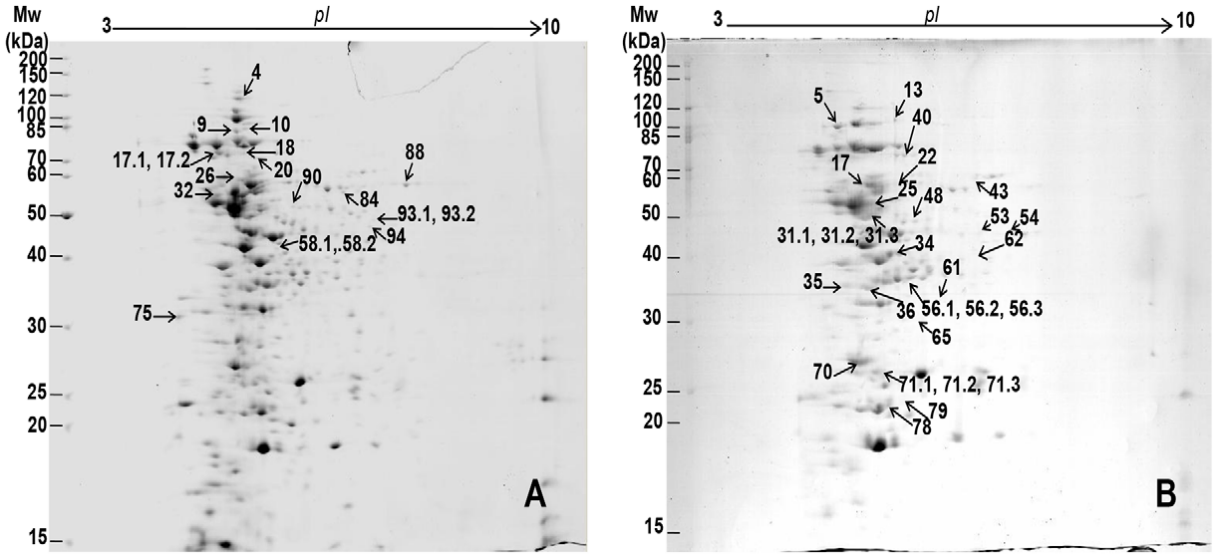
Two dimensional electrophoresis of the of cellular methicillin-resistant *S. aureus* proteomes. Protein extracts were prepared from rhodomyrtone-treated cells (A) and untreated control cells (B).

To preliminary address whether rhodomyrtone affected staphylococcal cell protein patterns, SDS-PAGE and Western immunoblotting were performed. Subinhibitory concentration of rhodomyrtone at 0.174 µg/ml (0.35MIC) was used to allow the bacterial growth at approximately 5×10^7^ cfu/ml after incubation for 18 h. Rhodomyrtone-treated staphylococcal cells exhibited several protein bands with increase or decrease in intensity, compared with the untreated controls. The expression of up- and down-regulated proteins was further assessed by immunoblot probed with mouse anti-*S. aureus* antisera. The protein patterns observed after immunoblotting clearly confirmed SDS-PAGE that both the extract and rhodomyrtone disturbed the profiles of immunogenic *S. aureus* cellular proteins in MRSA and *S. aureus* ATCC 29213 (data not shown). To characterize the effect of rhodomyrtone on whole-cell protein expression in MRSA, we further identified cellular proteins that appeared or disappeared following the exposure to 0.35MIC rhodomyrtone at 0.174 µg/ml in CAMHB for 18 h by 2DE in combination with mass spectrometry. Image analysis of 2DE patterns was performed by ImageScannerII and ImageMaster™ Software. Most of the protein spots were observed in acidic range as illustrated in Figures 2A and 2B. The mapping of cellular protein expression in MRSA revealed distinct alterations between the two proteomes. A total of 380 MRSA protein spots were observed in the cultures after treatment with rhodomyrtone while 301 spots were detected in cells grown in compound-free cultural media. Of these, 203 spots were shared (unchanged expression) between the two protein profiles. The investigation was focused on the more/less intense proteins. Of the overexpressed or downregulated protein spots in rhodomyrtone-treated MRSA, 18 proteins that presented only in rhodomyrtone treated-sample and 28 proteins that disappeared after the treatment were the proteins of interest. The presence or the absence of protein spots in the treated MRSA proteomes were denoted in Figures 2A and 2B. Protein spot number, protein annotation, functional category (NCBI database) are listed in Tables 1 and 2. The potential roles of these proteins are discussed below.

**Table 1:** Identification of *Staphylococcus aureus* cellular proteins presented after rhodomyrtone treatment.

Spot no.	Accession no.	Identified proteins	Mw	*pI*	Mascot	Sequence
			(KDa)		score	coverage (%)
Amino acid metabolism						
18	gi|21283809	D-fructose-6-phosphate amidotransferase	65925	4.93	460	17
20	gi|15925117	CTP synthetase	60297	5.00	191	8
94	gi|15924727	bifunctional 3-deoxy-7-phosphoheptulonate	40709	5.83	266	16
		synthase/chorismate mutase				
Carbohydrate metabolism						
4	gi|15924340	aconitate hydratase	99136	4.80	139	21
17.1	gi|15925529	pyruvate oxidase	63932	6.88	332	14
88	gi|15925597	malate:quinone oxidoreductase	56135	6.12	701	34
Cell wall metabolism						
58.1	gi|15925445	endo-1,4-*β*-glucanase	39212	5.21	326	16
Cell division						
84	gi|15924241	tRNA (uracil-5-)-methyltransferase Gid	48515	5.57	1012	40
Cofactor metabolism						
90	gi|15923960	coenzyme A disulfide reductase	49374	5.28	453	21
93.2	gi|15924201	pantothenate metabolism flavoprotein	44171	5.68	60	4
Energy metabolism						
26	gi|15925095	F0F1 ATP synthase subunit α	54608	4.91	595	18
Nucleotide metabolism						
10	gi|15924264	polynucleotide phosphorylase/polyadenylase	77428	4.89	1533	42
Protein degradation						
9	gi15925538	ATP-dependent Clp proteinase chain	77926	4.83	1256	33
Transcription						
32	gi|15924256	transcription elongation factor NusA	43787	4.60	267	14
75	gi|15924518	elongation factor P	20541	4.75	263	23
Transport system membrane						
17.2	gi|15924074	phosphoenolpyruvate-protein phosphatase	63394	4.62	266	11
58.2	gi|15925155	ATP-binding protein Mrp-like protein	38334	5.29	86	3
93.1	gi|15925438	glycine betaine/carnitine/choline ABC transporter	46463	5.93	108	5

**Table 2:** Identification of *Staphylococcus aureus* cellular proteins disappeared after rhodomyrtone treatment.

Spot no.	Accession no.	Identified proteins	Mw	*pI*	Mascot	Sequence
			(KDa)		score	coverage (%)
Amino acid and energy metabolisms						
22	gi|15924300	glutamine-ammonia ligase	51108	5.09	137	5
34	gi|49484831	ornithine carbamoyltransferase	37867	5.15	42	37
43	gi|57651929	glycine dehydrogenase subunit 2 (decarboxylating)	54861	5.70	65	19
48	gi|156978879	2-amino-3-ketobutyrate coenzyme A ligase	42935	5.14	79	43
53	gi|21283381	alanine dehydrogenase	40080	5.58	152	13
56.1	gi|49242958	carbamate kinase	35425	5.36	1086	35
56.2	gi|3892892	arginase	33170	5.29	165	8
61	gi|15925324	formimidoylglutamase	34605	5.35	53	7
62	gi|15923503	cysteine synthase (*O*-acetylserine sulfhydrylase)-like protein	33012	5.39	289	40
65	gi|49484892	putative *N*-acetyltransferase	30603	5.26	299	38
Carbohydrate metabolism						
17	gi|15924086	dihydrolipoamide dehydrogenase	49592	4.95	143	27
36	gi|49484802	fructose-1,6-bisphosphate aldolase	32893	4.96	46	28
40	gi|15924687	pyruvate kinase	63291	5.23	523	13
54	gi|221137774	glyceraldehyde 3-phosphate dehydrogenase 2	37126	6.07	137	9
56.3	gi|15925514	D-lactate dehydrogenase	39297	5.35	57	2
70	gi|15923764	triosephosphate isomerase	27417	4.81	71	39
Cell wall biosynthesis and metabolism						
13	gi|21282665	autolysin	137323	9.61	567	8
31.2	gi|15924634	GTPase ObgE	47263	5.05	190	11
71.2	gi|4948245	cell wall biosynthesis protein ScdA	25672	5.09	378	30
Lipid metabolism						
31.1	gi|21282595	3-oxoacyl- synthase	43883	5.03	1125	49
Cell division						
25	gi|15924176	cell division protein FtsZ	43135	4.74	523	32
Oxidative stress						
78	gi|15924543	superoxide dismutase	22697	5.08	221	18
Pyrimidine metabolism						
31.3	gi|15924191	dihydroorotase	46742	5.06	114	4
Protein degradation and Stress response						
5	gi|15924570	DnaK protein	66378	4.65	538	17
79	gi|15923758	ATP-dependent Clp protease proteolytic	21558	5.13	76	11
		subunit-like protein				
Surface antigen and virulence factor						
71.3	gi|4948478	immunodominant antigen A	24198	6.11	189	15
Others						
35	gi|49292908	putative hydrolase	30956	4.76	49	29
71.1	gi|4126674	hypothetical protein	22954	5.21	414	33

The presence of rhodomyrtone in MRSA cultures remarkably induced the expression of some general metabolic pathway related proteins based on their functions. Three proteins including D-fructose-6-phosphate amidotransferase, CTP synthetase, and bifunctional 3-deoxy-7-phosphoheptulonate synthase/chorismate mutase in amino acid metabolism and three proteins involved in carbohydrate metabolism such as aconitate hydratase, pyruvate oxidase, and malate:quinone oxidoreductase were identified. Two other proteins including coenzyme A disulfide reductase and pantothenate metabolism flavoprotein that related in cofactor metabolism were noted.

F0F1 ATP synthase subunit α was induced after treatment. The protein is partly embedded in the cell membrane and required for the regeneration of ATP from during the transfer of protons down the electrochemical gradient [24]. Protein members of chromosome partition, cell wall metabolism, and cell division such as endo-1, 4-*β*-glucanase and tRNA (uracil-5-)-methyltransferase Gid also up-regulated. Increase in the expression of three membrane transport systems-related proteins including phosphoenolpyruvate-protein phosphatase, ATP-binding protein Mrp-like protein, and glycine betaine/carnitine/choline ABC transporter, an osmoprotectant transport system permease protein involves in osmotic adjustment in bacteria [25], were observed.

Polynucleotide phosphorylase/polyadenylase (PNPase) was also up-regulated in rhodomyrtone-treated MRSA. This is an enzyme conserved in both bacteria and eukaryotic organelles that play roles in catalyzation of the phosphorolysis of RNA, releasing nucleotide diphosphates, and the reverse polymerization reaction. PNPase seems also to be participated in the control of several processes such as mRNA decay [26], cold shock response [27], and the post-transcriptional modification [28] of mRNAs in a variety of prokaryotes. The induction of transcription related proteins including transcription elongation factor NusA and elongation factor P, which are associated in protein biosynthesis were noted after the treatment. The bacteria may utilize these up-regulated proteins to compensate for the decrease in cell metabolic activities and to provide more efficient response to high-osmolarity stress due to rhodomyrtone challenge.

Rhodomyrtone inhibited the synthesis of six proteins functioning in glycolysis metabolic pathway such as fructose-1, 6-bisphosphate aldolase, pyruvate kinase, glyceraldehyde 3-phosphate dehydrogenase 2, D-lactate dehydrogenase, dihydrolipoamide dehydrogenase, and triosephosphate isomerase. Some of absent proteins have been reported to be participated with chromosome partition such as GTPase ObgE [29], cell wall biosynthesis, cell division, and cell wall turnover including cell wall biosynthesis protein ScdA [30], tubulin-liked FtsZ [31], and autolysin [32].

In bacteria, the of the cell wall is composed of the cross-linked polymer peptidoglycan, also known as murein. Peptidoglycan architecture consists of a disaccharide backbone built of alternating *β*-1-4-*N*-acetylglucosamines and *N*-acetylmuramic acids [33]. In *S. aureus*, the tetrapeptide chains allow the formation of peptide cross-bridge including L-alanine, D-isoglutamine, L-lysine, and D-alanine, which are attached to the *N*-acetylmuramic acid. This cross-linking of peptide cross-bridge is made up of five glycines [33]. Bacteria with compact peptidoglycan produce cell wall hydrolase, *N*-acetyl-glucosaminidase, *N*-acetylmuramidase, and endopeptidase for cell division [34]. It has been observed that rhodomyrtone suppressed the expression of autolysin with bifunctional activities, *N*-acetylmuramyl-L-alanine-amidase and *N*-acetylglucosaminidase. The bacterial cell division machinery is triggered with a formation of Z ring which consists of polymers of FtsZ protein at the division site [35]. FtsZ is the bacterial tubulin homologue which plays a key role for the recruitment of other proteins to the cell division septum [36]. The reduction of the cell wall-related proteins (GTPase ObgE, ScdA, FtsZ) following treatment with rhodomyrtone likely contributes to the reduced rate of cell wall biosynthesis, cell division, and cell wall morphogenesis and turnover. Additionally, suppressed production of autolysin might be the result as the bacterial cells react to prevent the destruction of peptidoglycan by autolytic enzymes. However, many of the enzymes involved in cell wall synthesis were not covered by the 2DE approach. An important challenge for our further studies will be to validate distinct alterations and clarify changes in gene expression following rhodomyrtone treatment. Thus, DNA microarray experiments would be extremely helpful to study effects on the transcription of genes involved in these processes, to provide more insight in potential mode of actions of rhodomyrtone.

A number of enzymes related in metabolism of some amino acids such as ornithine carbamoyltransferase and arginase in arginine biosynthesis, alanine dehydrogenase in alanine biosynthesis, glycine dehydrogenase subunit 2 (decarboxylating) and 2-amino-3-ketobutyrate coenzyme A ligase in glycine, serine, and threonine biosynthesis, formimidoylglutamase in histidine biosynthesis, and glutamine-ammonia ligase in glutamine biosynthesis were down-regulated. Other enzymes in several amino acid and energy metabolism were down regulated by subinhibitory concentration of rhodomyrtone were (i), cysteine synthase (*O*-acetylserine sulfhydrylase)-like protein which related with three metabolic pathways including cysteine, seleno amino acid, and sulfur metabolism; (ii), carbamate kinase in four metabolic pathways including purine, glutamate, arginine and proline, and nitrogen metabolism; and (iii), putative *N*-acetyltransferase. The reduction of alanine, glycine, and glutamine expression in rhodomyrtone-treated MRSA may be associated with the decrease in protein and peptidoglycan synthesis since those amino acid are crucial in both biological processes [37], [38]. Particularly, the lower glycine content which is an amino acid composition of peptidoglycan cross-bridges might excite the aberrant cell septum formation and retard cell division [39].

Other proteins involved in protein degradation, oxidative stress, putative hydrolase, and hypothetical protein were also inhibited after treatment. The stress response proteins were evidenced by the suppressing of the expression of heat-shock proteins including DnaK, ATP-dependent Clp protease proteolytic subunit-like protein, and oxidative stress-related protein such as superoxide dismutase. The proposed stress-related protein obviously reflected an adaption of the bacteria to survival under stress condition. In *S. aureus*, Clp proteins play significant roles in the regulation of many cellular functions such as replication of DNA, control of gene expression, heat stress tolerance, protein degradation, and protein folding as molecular chaperones [40], [41]. In addition, they also related to various biological processes such as cell division, development of sporulation, genetic competence, and quality control of protein translation [42], [43]. Clp proteinase are composed of two distinct subunits, proteolytic part is assembled with ATPase subunit into a multimeric structure. In *E. coli*, Clp protease is known to play a role in cytoplasmic quality control and participate in numerous regulatory mechanisms that are important in non-growing or slow-growing cells [44]. In this study, ATP-dependent Clp protease proteolytic subunit-like protein (ClpP, proteolytic subunit) was suppressed whereas ATP-dependent Clp proteinase chain (ClpB, ATPase subunit) was induced. In *E. coli*, ClpB has a crucial function in cell survival at high temperatures [45] and participates in *de novo* protein folding in mildly stressed [46]. Our data suggested that MRSA responded to stress and degradation of many intracellular proteins after exposure to rhomyrtone at subinhibitory concentration. The compound also inhibited the expression of immunodominant antigen A, a virulence factor which elicits immune response during septicaemia [47].

### Effect of rhodomyrtone on *S. aureus* cell morphology

**Figure 3:**
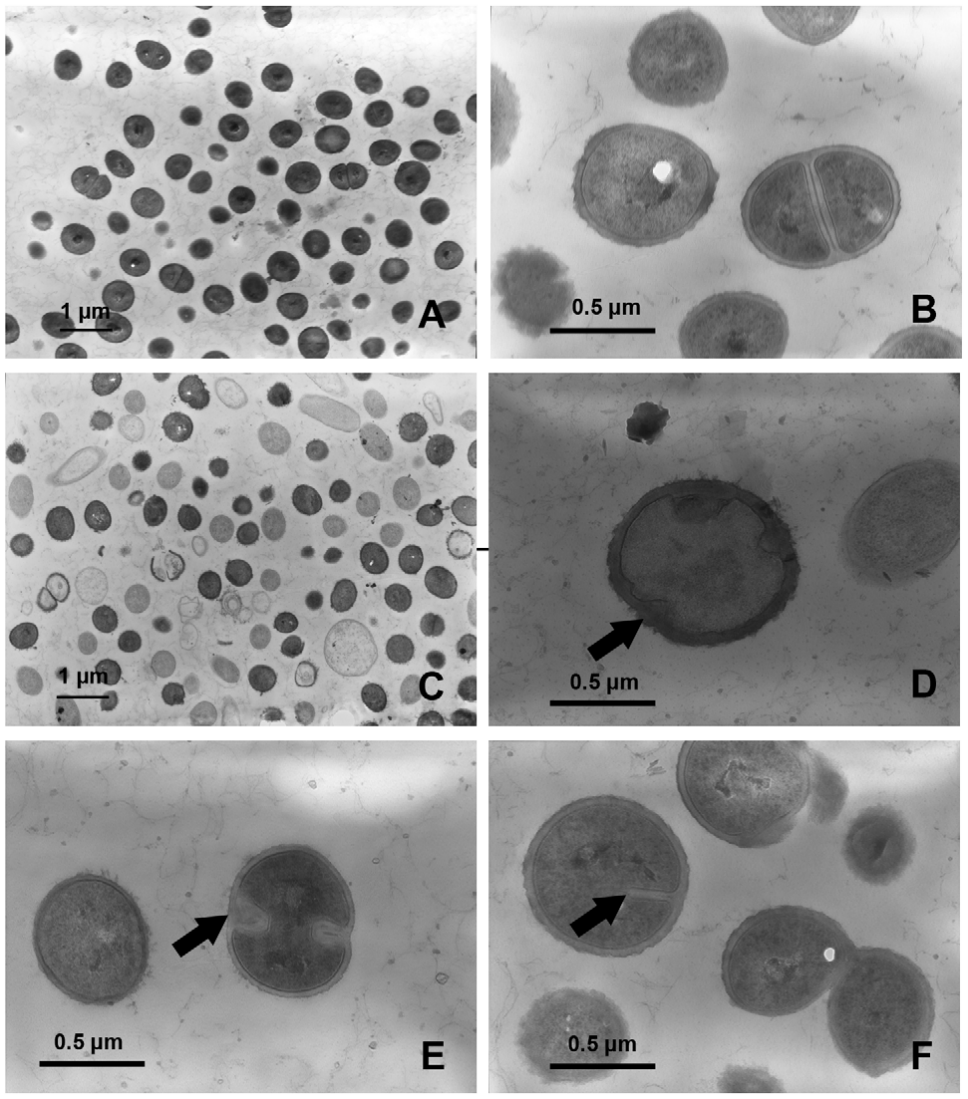
Transmission electron microscopy demonstrating the effects of rhodomyrtone on methicillin-resistant *S. aureus* NPRC 001R morphology and ultrastructure. The bacteria were incubated in CAMHB for 18 h, media containing 0.174 µg/ml of rhodomyrtone (C, D, E, and F) and untreated control cultures (A and B). Scale bars = 1 µm (A and C) and 0.5 µm (B, D, E, and F), respectively.

**Figure 4:**
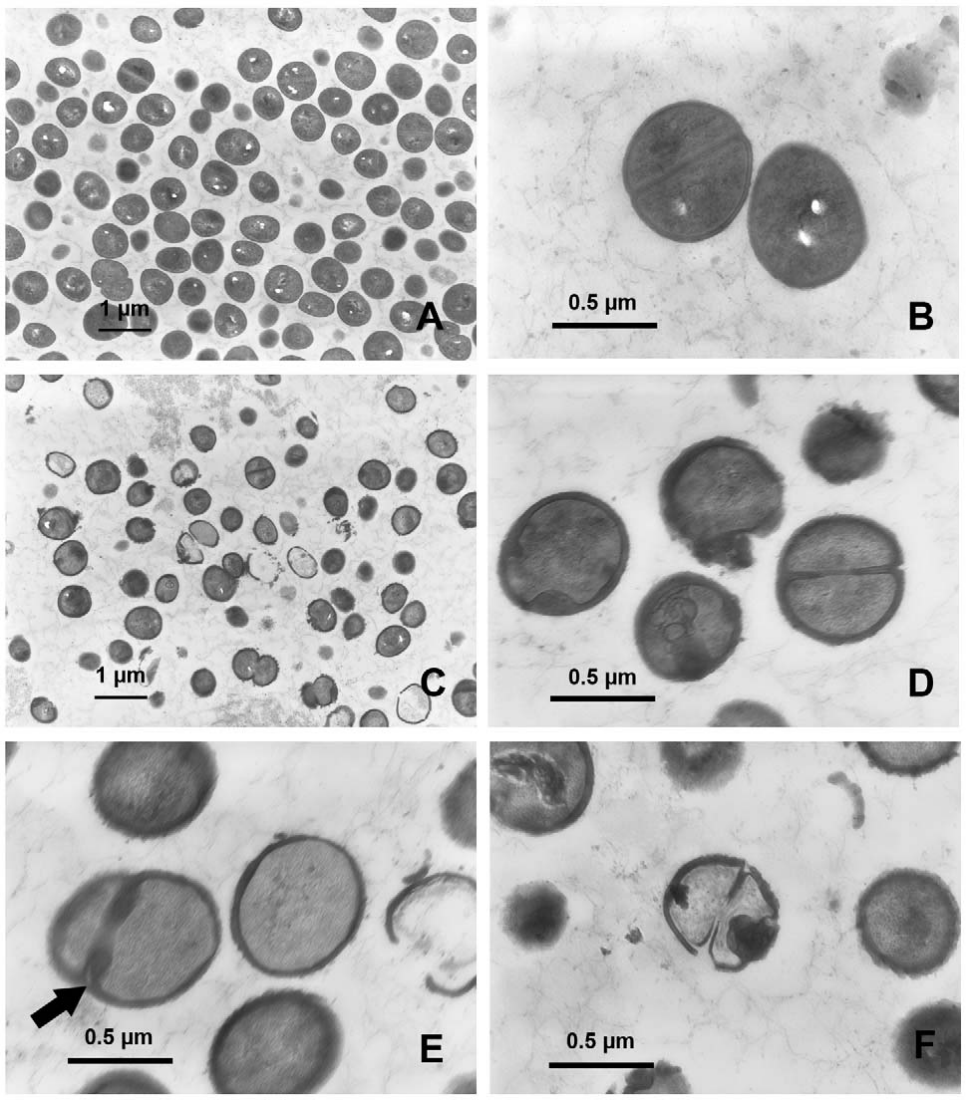
Transmission electron microscopy demonstrating the effects of rhodomyrtone on *S. aureus* ATCC 29213 morphology and ultrastructure. The bacteria were incubated in CAMHB for 18 h, media containing 0.174 µg/ml of rhodomyrtone (C, D, E, and F) and untreated control cultures (A and B). Scale bars = 1 µm (A and C) and 0.5 µm (B, D, E, and F), respectively.

To elucidate the physiological effects of rhodomyrtone against *S. aureus*, transmission electron microscopy was utilized. As illustrated in Figure 3 and Figure 4, the micrographs clearly demonstrated that the growth of both MRSA and *S. aureus* ATCC 29213 in media containing rhodomyrtone at 0.174 µg/ml for 18 h generated profound changes in both *S. aureus* cell morphology and ultrastructure. Almost all rhodomyrtone-treated cells were obviously affected on the cell envelope, cytoplasmic contents, cell ultrastructure, and cell separation process. Interestingly, we found that rhodomyrtone induced cell wall deformation (Figure 3C), thickened cell wall (Figure 3D, arrow), and thickened septa with irregular features (Figures 3E, 3F and 4E, arrows). Increasing in staphylococcal cell wall thickness has been previously reported due to the mechanism of actions of some antibiotics such as chloramphenicol [34], erythromycin [48], penicillin [34], and vancomycin [49]. Furthermore, the arc-like shape of fibrillar wall material occurring from turning of compressed cross wall and septal formation (Figures 3E and 4E, arrows) were also observed in rhodomyrtone exposed cells. This effect of the compound was similar to staphylococcal cells grown in the presence of penicillin at a concentration of 0.05 µg/ml [34]. After treatment with low dose of penicillin, staphylococcal cells always affect the penicillin binding protein which is considered to be located in the splitting system [50]. Cell separation system is inhibited and only the fibrillar wall material is organized instead of a compact cross wall. The results might suggest that cells with thickened walls may represent a defensive response to rhodomyrtone. Cells with broken wall, protoplast, cell wall shreds, and cell debris were generally found following rhodomyrtone treatment (Figures 3C, 3F and 4C, 4D, 4F). Indeed, various atypical size and cell shape alterations, for example, irregular, swollen, oval-shaped, and defective cells were obviously noticeable, especially in the treated MRSA (Figure 3C), while these aberrations were not observed in the untreated control cells (Figure 4C). Overnight exposure of *S. aureus* ATCC 29213 to rhodomyrtone resulted in almost complete lysis of intact bacteria to cellular debris (Figure 4E). In this study, rhodomyrtone was found to cause cell wall and cytoplasmic membrane damage in *S. aureus*. Meanwhile, it exhibited only insignificant morphological effects to *Streptococcus mutans* using phase contrast microscopy, indistinct enlarged cells have been detected after treatment with 5MIC rhodomyrtone for 20 h [15].

In conclusion, our results from proteomic analysis demonstrated consequences of the alterations of several cellular protein expressions on MRSA and *S. aureus* cultures containing rhodomyrtone at subinhibitory concentration. Most differentially expressed MRSA proteins belong to the functional categories such as amino acid metabolism, carbohydrate metabolism, and cell wall biosynthesis, suggesting that these might be the proteins affected most by rhodomyrtone. Transmission electron micrographs which revealed the morphological and ultrastructural alterations of the treated cells supported the protein analysis data, indicating that cell exposure to subinhibitory concentration of rhodomyrtone for 18 h might involve damage of cell wall turnover or membrane-related proteins.

The data reported in this paper support the understanding on prominent antibacterial effects of rhodomyrtone that relate to cell wall biosynthesis, cell membrane function, and cell division of MRSA. The information shed light on the antimicrobial activities of a new interest natural compound which might be a potential candidate for a novel therapeutic agent for the treatment of MRSA infections.

## Materials and Methods

### Preparation of ethanolic extract and rhodomyrtone

Dried leaves of *R. tomentosa* were extracted with 95% ethanol as has been described previously [15]. Purification protocol and the structure of rhodomyrtone, a major bioactive principle, have been previously published by our research group [15], [51]. Purity of rhodomyrtone has been confirmed by referring to nuclear magnetic resonance (NMR) and mass spectrometry (MS) reference [12], [51].

### Bacterial strains and growth conditions

MRSA NPRC 001R, a common endemic isolate with *mecA* gene obtained from Hat Yai hospital, Thailand [52] and *S. aureus* ATCC 29213 were used throughout this study. The bacterial strains were stored as a 20% glycerol stock at −80°C until required. Bacteria were precultured overnight from glycerol stock on Luria-Bertani agar. The bacteria were inoculated into 2 ml of trypticase soy broth at 37°C with constant shaking to mid-exponential phase growth.

### Determination of minimal inhibitory concentration (MIC)

MICs of the crude extract and rhodomyrtone against *S. aureus* were carried out by a modified broth microdilution method recommended by Clinical Laboratory Standardization Institute (CLSI) guideline [53]. Two fold-serially dilution in a 96-well microtiter plate of the extract and rhodomyrtone was prepared to obtain final concentration ranged from 0.24 to 500 µg/ml and 0.0625 to 128 µg/ml, respectively. Briefly, an exponential phase growth of *S. aureus* was diluted to a density of 10^8^ cfu/ml in CAMHB (Difco, Detroit, MI). The bacterial suspension was then added to each well to provide a final inoculum density of 5×10^5^ cfu/ml. After incubation at 37°C for 16–18 h, the bacterial suspension in each well was measured at OD_600 nm_ against a blank well (the broth containing 1% dimethyl sulphoxide, DMSO) with an ELISA reader (Labsystems). The MIC was defined as the lowest concentration of the agent that completely inhibited the bacterial growth. All experiments were carried out in triplicate.

### Time-kill study

Time-kill kinetic studies of the rhodomyrtone were performed in cation-adjusted Mueller Hilton broth (CAMHB). An overnight culture was used to inoculate in CAMHB containing rhodomyrtone at 0.125, 0.25, 0.5, 1, and 2MIC. The initial inoculum was approximately 10^8^ cfu/ml. A culture containing 1% DMSO and no rhodomyrtone was included in each assay as a growth control. Sampling was done at 0, 1, 2, 3, 4, 5, 6, 8, 18, and 24 h. Viable bacteria were determined by plate counts on Mueller Hilton agar. The activity of rhodomyrtone was determined by plotting log_10_ colony count (cfu/ml) against time. Values shown are the means of duplicate determinations from the experiments repeated three times.

### Bacterial cell protein preparation

Staphylococcal cells were collected by centrifugation at 10,000×*g* at 4°C for 20 min. The culture supernatant was removed and the pellets were kept at -80°C until used. To prepare cellular proteins, the lysate was washed twice with 0.85% (w/v) NaCl and resuspended in phosphate buffer saline (PBS). The cells were agitated by a 750-Watt Ultrasonic processor at 30% amplitude for 15 min on ice, pulse durations of 9 sec on and 9 sec off. The lyzed cells were centrifuged at 10,000×*g* at 4°C for 20 min to remove the cellular debris and aggregate. Protein concentrations were quantified by Bradford protein assay kit (Bio-Rad, Hercules, CA) according to the manufacturer's instruction. Bovine serum albumin (BSA) was used as protein standard. The supernate containing solubilized proteins was used directly or stored at −80°C until required.

### SDS-PAGE

The protein samples were suspended in SDS sample buffer (62.5 mM Tris (pH 6.8), 2% (w/v) SDS, 10% (v/v) glycerol, 5% (v/v) mercaptoethanol, 0.05% (w/v) bromphenol blue) and heated at 100°C for 3–5 min prior to loading. Each 200 µg protein (5–10 µl) was applied on acrylamide gels on Mighty® Small II SE250 gel apparatus (Hoefer® Pharmacia Biotech). Proteins were separated based on their molecular weight by electrophoresis at 25 mA through a stacking gel (4% acrylamide) and a separating gel (12.5% acrylamide). The gels were then stained by overnight incubation with gentle agitation in staining solution (0.5% (w/v) Coomassie Brilliant Blue G-250 in 20% (v/v) methanol, 2% (v/v) *O*-phosphoric acid, 8% (w/v) ammonium sulphate). Prestained protein ladder (Bio-Rad) was used for SDS-PAGE molecular weight markers.

### Western blot analysis

The method used was a modification of that described by Towbin *et al*. [54]. The samples were run on 12.5% polyacrylamide gels and transferred onto nitrocellulose membrane (Amersham bioscience) at 100V for 1 h in transfer buffer (25 mM Tris-HCl (pH 8.3), 192 mM glycine, 10% (v/v) methanol) using a Transblot unit (Bio-Rad). Nitrocellulose sheet was blocked by incubating for 30 min in 5% (w/v) skim milk powder in PBST (PBS with 0.05% (v/v) Tween-20), and followed by incubation in mouse anti-*S. aureus* antiserum (1∶3,000 dilution in PBST containing 0.2% (w/v) BSA and 0.2% (w/v) gelatin) at 4°C for 1–16 h. The antiserum was removed and the nitrocellulose membrane was washed three times for 5 min in PBST with gentle agitation. Horse radish peroxidase-conjugated goat anti-mouse (Southern biotechnology) was added at a dilution of 1∶3,000 in PBST containing 0.2% (w/v) BSA and 0.2% (w/v) gelatin, and incubated for 1–2 h with gentle agitation at room temperature. The sheet was then washed four times in PBST and equilibrated in 67 mM phosphate buffer (pH 7.6). Antigen-antibody complexes were detected by the addition of color detection solution (67 mM phosphate buffer (pH 7.6) containing of 0.2% (w/v) 2,6-dichloroinophenol and 0.03% hydrogen peroxide) and incubated in the dark. The color reaction was stopped with deionized water.

### Preparation of 2D-gel samples

The bacterial cell samples were prepared from four liter cultures in the presence or the absence of subinhibitory concentration of rhodomyrtone (0.174 µg/ml) after incubation for 18 h. Untreated control cultures were grown in MHB containing 1% DMSO. The pellets were then resuspended in standard cell lysis buffer (30 mM Tris, 2 M thiourea, 7 M urea, 4% CHAPS) containing protease inhibitors (Roche Diagnostics, Mannheim, Germany). Each sample was subjected at 30% amplitude, 2 sec pulse-on, 2.5 sec pulse-off, for a total of 5 min in an ice water bath. After sonication, the lyzed cells were centrifuged and the supernatant was transferred to a new tube. The pH of the homogenate was adjusted to 8.5 by slow and careful addition of 50 mM sodium hydroxide. The mixtures were stored in aliquots at −70°C until required. The proteins were cleaned with 2D-Clean-up kit (Amersham Biosciences) to eliminate detergents, salts, lipids, phenolics, and nucleic acids. After the treatment, the samples were solubilized with DeStreak Rehydration solution (Amersham Biosciences). Protein concentrations were quantitated by 2D-Quant kit (Amersham Biosciences) with BSA as the protein standard before subjecting to isoelectric focusing.

### 2D-gel electrophoresis

To separate the cellular proteins in first dimension, the samples (200–500 µg) in DeStreak Rehydration solution containing 1% pH range of 3–10 immobilized pH gradient (IPG) buffer (Amersham Biosciences) were applied onto the center of a slot in the 7 or 18 cm strip holder of the IPG phor (GE Healthcare Biosciences). The IPG strip (pH 3–10, non linear) (GE Healthcare Biosciences) was placed into the strip holder containing the sample. The PlusOne™ DryStrip Cover Fluid (Amesham Biosciences) was overlaid onto each IPG strips to prevent evaporation and urea crystallization. The strip holder was then placed into the Ettan IPG Phor Electrofocusing System (Amersham Biosciences) and the IPG strip was allowed to rehydrate at 20°C for 12 h. The isoelectric focusing parameters were as followed; the voltage was set at 300 V (Volt) until 200 Volt-hours (Vh), 1,000 V until 300 Vh, 5,000 V until 4,000 Vh, and 5,000 V until 2,000 Vh.

For the second dimension gel electrophoresis, each electrofocused-IPG strip was equilibrated in 5 ml SDS-equilibration buffer (50 mM Tris-HCl, pH 8.8, 6 M urea, 30% glycerol, 2% SDS, and 0.002% bromphenol blue) containing 50 mg dithiothreitol (DTT) for 15 min at room temperature. The strip was then placed in 10 ml of the equilibration buffer containing 250 mg iodoacetamide (IAA) for 15 min, washed by 1x electrode buffer, and placed onto a 1 mm thick 12.5% polyacrylamide gel (10×11 cm^2^) precasted in HoeferTMDual Gel Caster (Amersham Biosciences). The second dimensional gel was carried out at 25 mA/gel until the tracking dye reached the lower edge of the gel. After electrophoresis, the gel was subjected to colloidal Coomassie brilliant blue staining. The molecular weight was estimated according to the internal running calibration marker.

### Image analysis

For visualization, gels were briefly rinsed with 1% acetic acid to reduce background. The comparative analysis using ImageScannerII and ImageMaster™ Software (Amersham Biosciences) was performed to detect differences in protein productions between gel images of protein profiles obtained from rhodomyrtone-treated cell proteins and the untreated cell proteins. Protein changes in 2D-gels were analyzed from three independent experiments (biological replicates) and similar protein patterns were observed among the gels. Unchanged expression of protein spot was considered if protein intensity was less than two-fold (*p*>0.05). Overexpressed or downregulated protein spots occurred after rhodomyrtone treatment were excised recorded from representative gels. Selected spots that presented only in rhodomyrtone-treated *S. aureus* samples and those that disappeared after the treatment were individually excised from the respective Coomassie dye stained-gels.

### In-gel digestion and peptide extraction

Protein spots were destained using washing solution containing 50% methanol and 5% acetic acid in ultra deep water at 25°C for 18 h. Gel plugs were rinsed a few times in a period of 2–3 h with fresh washing solution. The gel pieces were dehydrated with 100% acetonitrile (ACN) and reduced with 10 mM DTT in 10 mM ammonium bicarbonate at room temperature for 1 h. For alkylation, 100 mM IAA in 10 mM ammonium bicarbonate was added to the gel plugs and incubated at room temperature for 1 h in the dark. After alkylation, the gel pieces were dehydrated twice with 100% ACN for 5 min. To perform in-gel digestion of proteins, 10 µl of trypsin solution (10 ng/µl trypsin in 50% ACN/10 mM ammonium bicarbonate) were added to the gels and incubated at room temperature for 20 min. After incubation, 20 µl of 30% ACN was added to keep the gels immersed throughout digestion. The gels were incubated at 37°C for a few hours or overnight. To extract peptide digestion products, 30 µl of 50% ACN in 0.1% formic acid was added into the gels, and the gels were then incubated at room temperature for 10 min in a shaker. Extracted peptides were collected and pooled. The peptides were dried by vacuum centrifuge and kept at −80°C for further mass spectrometric analysis.

### Matrix assisted laser desorption ionization–time of flight mass spectrometry (MALDI-TOF/MS)

Identification of interested protein spots was performed using MALDI-TOF/MS. The tryptic digests were spotted with α-cyano-4-hydroxy-cinnamic acid (Sigma-Aldrich, St.Louis, MO) matrix solution, and analyzed with an Ultraflex III MALDI-TOF/TOF MS (Bruker Daltonics, Bremen, Germany). Mass spectra were recorded in the positive-ion, delayed extraction mode. All spectra were acquired with 20 kV accelerating voltage, 150 ns delayed extraction time, and 900 Da low-mass gate. All spectra were externally mass calibrated with ProteoMassTM Peptide and Protein MALDI-MS Calibration Kit (Sigma-Aldrich).

### Liquid chromatography-mass spectrometry (LC-MS)

The protein digests from rhodomyrtone-treated and untreated MRSA were injected into Ultimate 3000 LC System (Dionex) coupled to ESI-Ion Trap MS (HCT Ultra PTM Discovery System [Bruker]) with electrospray at flow rate of 300 nl/min to a nanocolumn (Acclaim PepMap 100 C18, 3 µm, 100A, 75 µm id×150 mm). A solvent gradient (solvent A: 0.1% formic acid in water; solvent B: 0.1% formic acid in 80% acetonitrile) was run in 40 min.

### Database searching

All of the tandem mass spectra were analyzed against a public database (National Center for Biotechnology Information) or Swiss-Prot from MALDI fingerprint data by MASCOT (http://www.matrixscience.com). Protein identification was performed on the basis of statistically significant Mowse score (*p*<0.05).

### Transmission electron microscopic analysis

Overnight cultures grown in the presence or the absence of rhodomyrtone at 0.174 µg/ml were harvested by centrifugation at 10,000×*g* for 5 min. The cells were washed twice, resuspended in PBS, and fixed with 2.5% glutaraldehyde in 0.1 M cacodylate. The samples were then incubated at 4°C for at least 1 h. After incubation, the cells were recovered by centrifugation at 10,000×*g* for 5 min and washed twice with PBS. The pellets were fixed with 1% osmium tetraoxide and were then left at room temperature for 1 h. The samples were dehydrated with graded ethanol solutions such as 30% ethanol for 10 min, 50% ethanol for 10 min, 70% ethanol for 10 min, 90% ethanol for 10 min, and twice in 100% ethanol for 15 min. They were fixed in Epon 812 resin and left to polymerize for 3 days. Each sample was cut into thin slices, approximately 90 nm, with a glass knife, stained with uranyl acetate, and lead citrate on grids. Mophological and ultrastructural of the bacteria were observed and photographed by a Hitashi H7000 at 75 kV.
